# Supplementary material for: Mass cytometry reveals cellular fingerprint associated with IgE+ peanut tolerance and allergy in early life
Source: Nat Commun. 2020 Feb 27;11:1091. doi: 10.1038/s41467-020-14919-4 (PMC7046671; doi:10.1038/s41467-020-14919-4)
Supplement: Supplementary file 1 — Supplementary Data [file 41467_2020_14919_MOESM1_ESM.pdf]

Mass cytometry reveals cellular fingerprint associated with IgE+ peanut tolerance and allergy in early life

Neeland and Andorf et al.

Table S1. Mass cytometry antibody panel and staining volumes

	Surface Marker	Metal label	Vol (µl) / 70µl reaction
1	CD86	113In	1.4
2	CD19	142Nd	0.7
3	CD49b	143Nd	1.4
4	CD4	145Nd	0.7
5	CD8	146Nd	0.7
6	CD20	147Sm	0.7
7	CD38	148Nd	0.7
8	CCR4	149Sm	0.7
9	LAG3	150Nd	0.7
10	CD123	151Eu	0.7
11	CD45RA	153Eu	0.7
12	CD3	154Sm	0.7
13	CD28	155Gd	0.7
14	HLA-DR	157Gd	0.7
15	CD33	158Gd	0.7
16	CD11c	159Tb	0.7
17	CD14	160Gd	0.7
18	CXCR3	163Dy	0.7
19	CD127	165Ho	0.7
20	CD27	167Er	0.7
21	CCR7	169Tm	1.1
22	CD25	173Yb	0.7
23	CD56	176Yb	0.7
24	CD16	209Bi	0.7
	**Intracellular Marker**	**Metal label**	**Vol (µl) / 70µl reaction**
25	IL-4	144Nd	0.7
26	TNFα	152Sm	0.7
27	IFNγ	161Dy	0.7
28	CD69	162Dy	0.7
29	IL-17	164Dy	0.7
30	IL-2	166Er	0.7
31	CD40L	168Er	0.7
32	IL-10	171Yb	0.7

Table S2: % TNFα producing cells of parent population following PMA/ionomycin stimulation. Shown are median (mean, minimum - maximum).

Parent population	NA	PST	PA	total	p[$]
B cells	2.43 (2.86, 1.57-5.55)	2.22 (2.95, 1.1-5.73)	3.76 (3.65, 1.82-6.3)	2.75 (3.15, 1.1-6.3)	0.27
CD8 T cells	4.96 (5.75, 1.74-15.9)	4.92 (7.08, 1.77-20.3)	3.06 (3.95, 1.45-8.82)	4.67 (5.59, 1.45-20.3)	0.21
CD4 T cells	10.95 (12.01, 6.2-21)	8.29 (12.73, 4.96-36.8)	13.45 (16.15, 7.51-35.9)	11.65 (13.63, 4.96-36.8)	0.14
NK cells	31.9 (28.47, 9.24-47.6)	19.95 (21.83, 12.2-38.9)	28.45 (30.54, 15.3-50.9)	25.25 (26.95, 9.24-50.9)	0.45
Monocytes	9.13 (9.89, 1.08-19)	5.4 (7.82, 0.68-19.2)	12.45 (14.23, 1.69-42.9)	9.13 (10.65, 0.68-42.9)	0.18
pDCs	3.09 (3.23, 0-6.96)	3.48 (4.82, 0-17.7)	8.16 (8.98, 2.08-22.2)	4.54 (5.67, 0-22.2)	0.014[#]
mDCs	1.23 (1.23, 0.053-2.75)	0.77 (0.95, 0.023-2.9)	1.43 (1.29, 0.19-2.3)	1.22 (1.16, 0.023-2.9)	0.52

[$] p values by linear mixed effects model and χ^2 test between the three groups

[#] follow-up comparisons between two groups: NA – PST: p = 0.34, NA – PA: 0.0058, PST – PA: p = 0.037

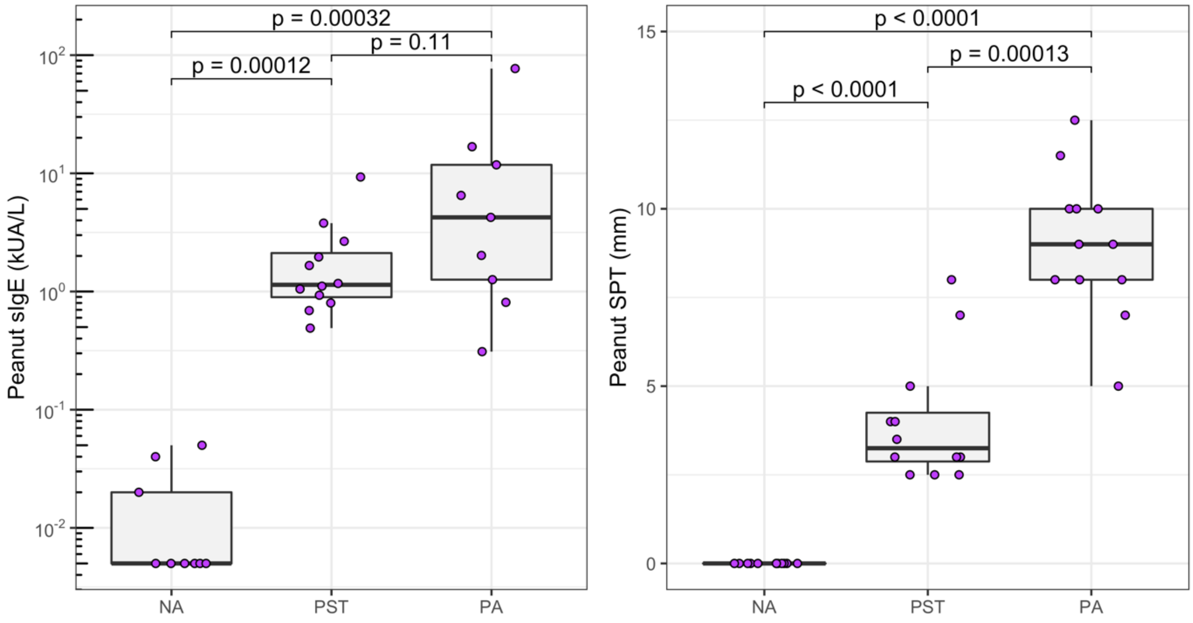

Fig. S1. Peanut sIgE and peanut SPT values for the three study groups (non-sensitized, non-food allergic (NA), peanut sensitised tolerant (PST) and peanut allergic (PA)) are shown. Peanut sIgE values at the lower detection limit of 0.01kUA/L were set to 0.005kUA/L. P values by Wilcoxon rank sum test. In the boxplots the medians are shown. The ‘hinges’ represent the first and third quartile. The whiskers are the smallest and largest values after exclusion of outliers (greater than the 75th percentile plus 1.5 times the IQR, or less than 25th percentile minus 1.5 times the IQR).

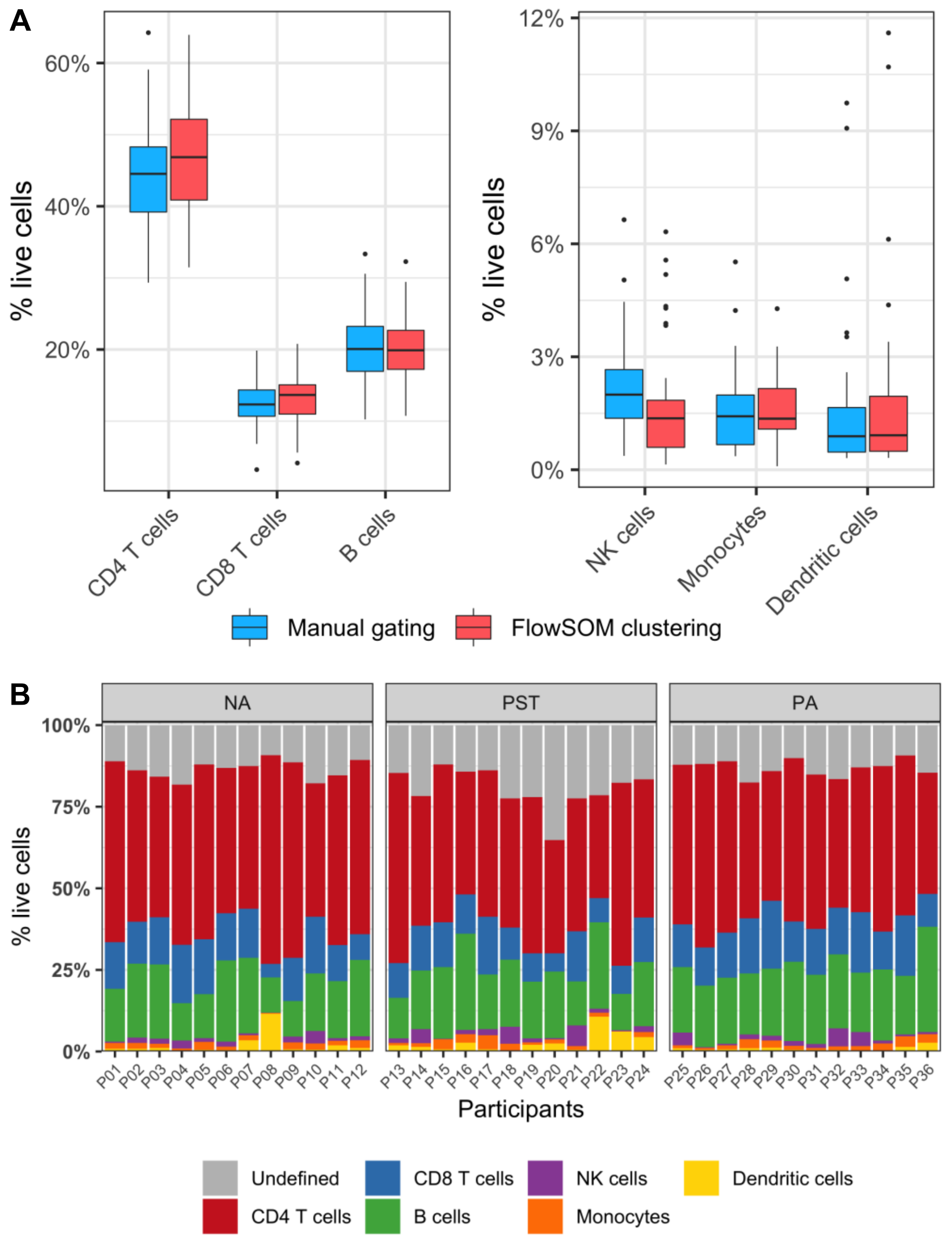

Fig. S2. Comparison of results from unsupervised clustering analysis (FlowSOM) and manual gating. (A) Frequencies of the major cell populations obtained by manual gating (blue) and FlowSOM clustering (red). **(B)** Frequencies of the major cell populations as determined by FlowSOM clustering for individual infants across the three study groups (non-sensitized, non-food allergic (NA), peanut sensitised tolerant (PST) and peanut allergic (PA)). In the boxplots the medians are shown. The 'hinges' represent the first and third quartile. The whiskers are the smallest and largest values after exclusion of outliers (greater than the 75th percentile plus 1.5 times the IQR, or less than 25th percentile minus 1.5 times the IQR).

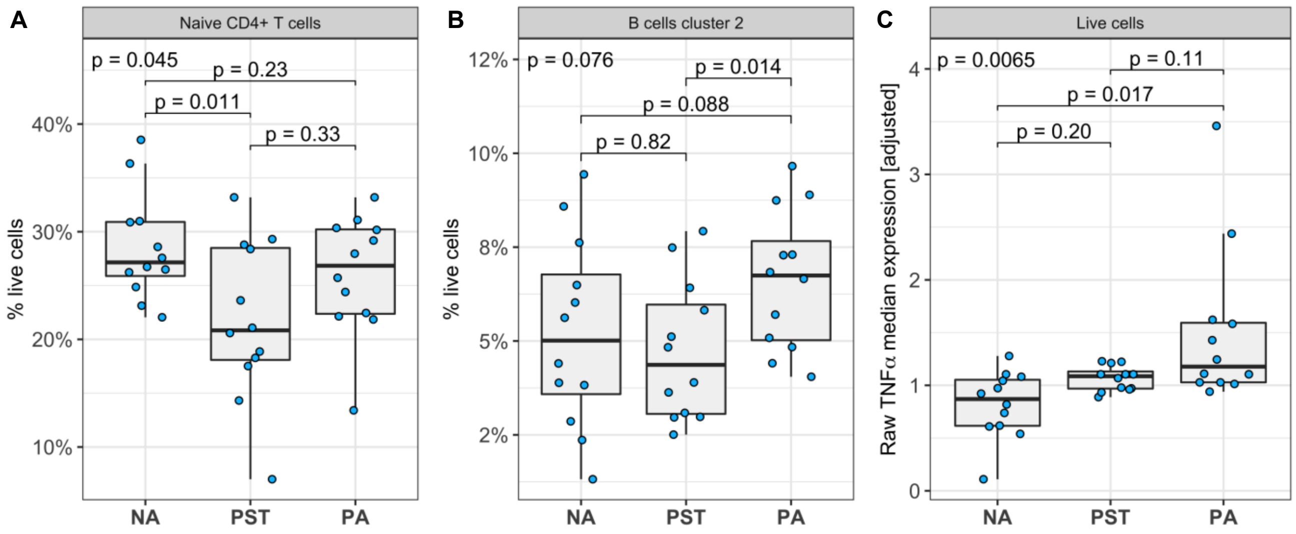

Fig. S3. (A+B) Statistically significant results obtained by unsupervised analysis are confirmed by manual gating analysis of the respective populations. **(C)** Significant differences in the frequency of TNFα$^+$ cells across all live, single cells between the three groups (non-sensitized, non-food allergic (NA), peanut sensitised tolerant (PST) and peanut allergic (PA)) are confirmed as change in median raw expression of TNFα between the three groups. Values were adjusted for batch before plotting. All p values were determined by χ^2 tests in linear mixed effects models. In the boxplots the medians are shown. The 'hinges' represent the first and third quartile. The whiskers are the smallest and largest values after exclusion of outliers (greater than the 75th percentile plus 1.5 times the IQR, or less than 25th percentile minus 1.5 times the IQR).

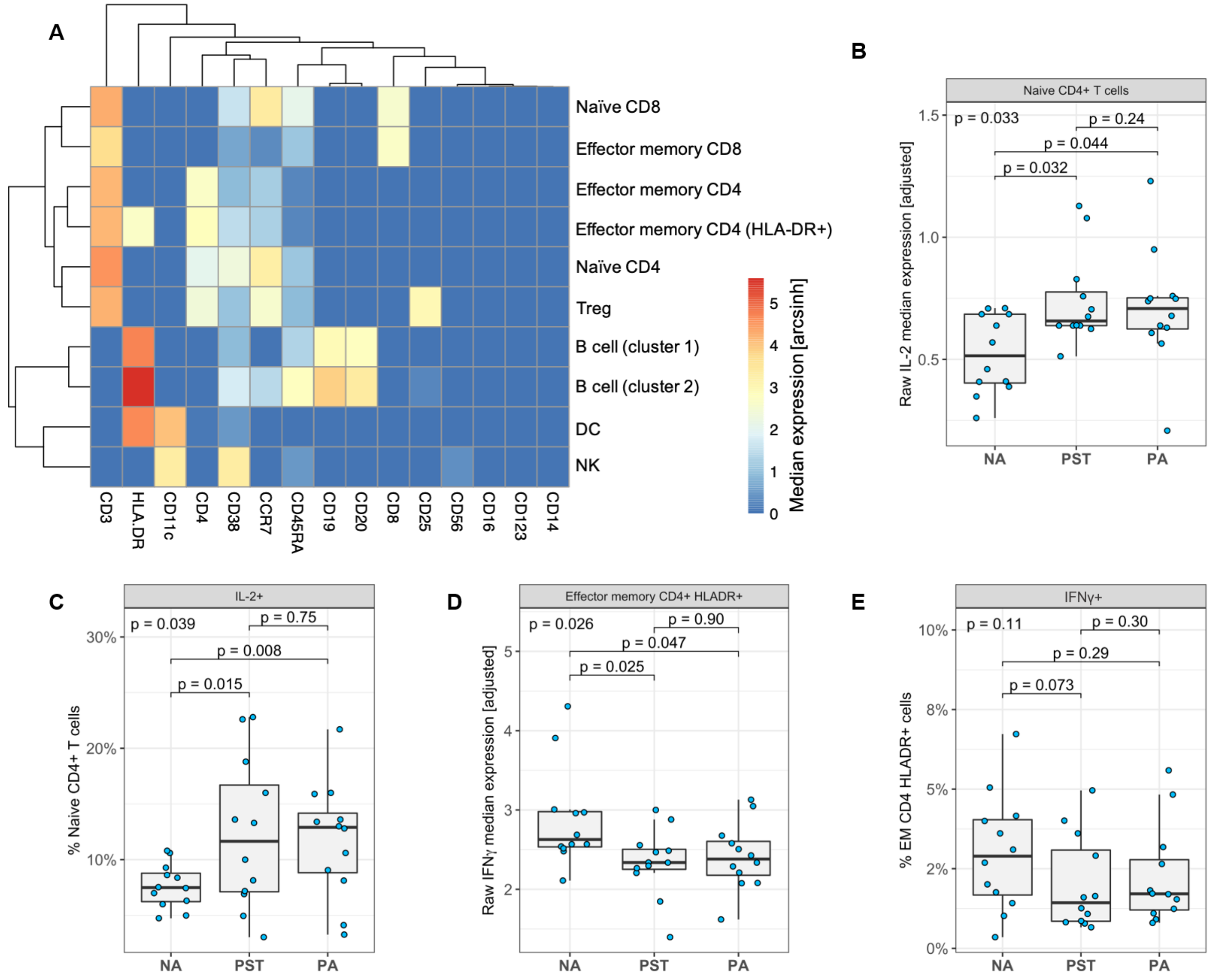

Fig. S4. FlowSOM analysis of PMA/ionomycin stimulated samples. (A) Ten clusters which could be assigned to known cell populations were detected, shown here as a heatmap of the median expression of lineage markers. **(B-C)** IL-2 expression in the naive CD4 T cell population as determined by manual gating, expressed as median IL-2 expression and as percentage of naïve CD4 cells in the three groups (non-sensitized, non-food allergic (NA), peanut sensitised tolerant (PST) and peanut allergic (PA)). **(D-E)** IFNγ expression in the effector memory (EM) CD4 T cell population expressing HLA-DR as determined by manual gating, expressed as median IL-2 expression and as percentage of EM HLADR$^+$ cells. All p values were determined by χ^2 tests in linear mixed effects models. Median expression values were adjusted for batch before plotting. In the boxplots the medians are shown. The 'hinges' represent the first and third quartile. The whiskers are the smallest and largest values after exclusion of outliers (greater than the 75th percentile plus 1.5 times the IQR, or less than 25th percentile minus 1.5 times the IQR).

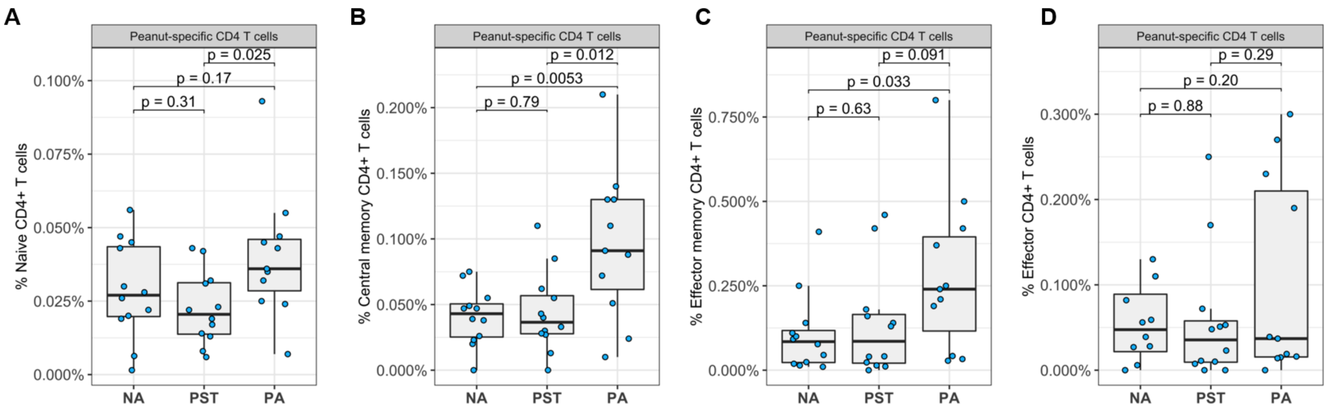

Fig. S5. Analysis of CD45RA and CCR7 expression in peanut-specific CD4 T cells following 24h peanut stimulation. **(A)** Frequency of naïve CD4 T cells ($CD45RA^{+}CCR7^{+}$) co-expressing CD40L and CD69 following peanut stimulation in the three groups (non-sensitized, non-food allergic (NA), peanut sensitised tolerant (PST) and peanut allergic (PA)) **(B)** Frequency of central memory CD4 T cells ($CD45RA^{-}CCR7^{+}$) co-expressing CD40L and CD69 following peanut stimulation. **(C)** Frequency of effector memory CD4 T cells ($CD45RA^{-}CCR7^{-}$) co-expressing CD40L and CD69 following peanut stimulation. **(D)** Frequency of effector CD4 T cells ($CD45RA^{+}CCR7^{-}$) co-expressing CD40L and CD69 following peanut stimulation. P values by F tests in linear models. In the boxplots the medians are shown. The 'hinges' represent the first and third quartile. The whiskers are the smallest and largest values after exclusion of outliers (greater than the 75th percentile plus 1.5 times the IQR, or less than 25th percentile minus 1.5 times the IQR).

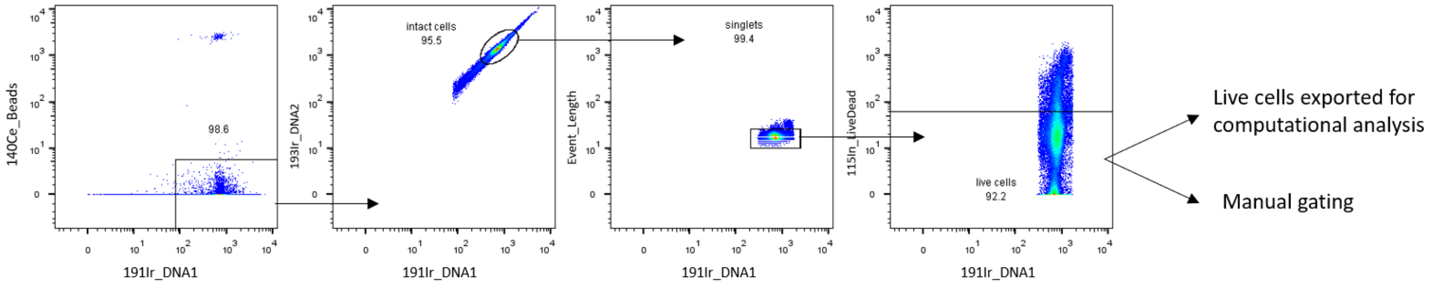

Fig. S6. Pre-processing of FCS files to remove normalisation beads and debris, and to select for live single cells. The FCS files containing only live cells were exported for computational analysis and manual gating.

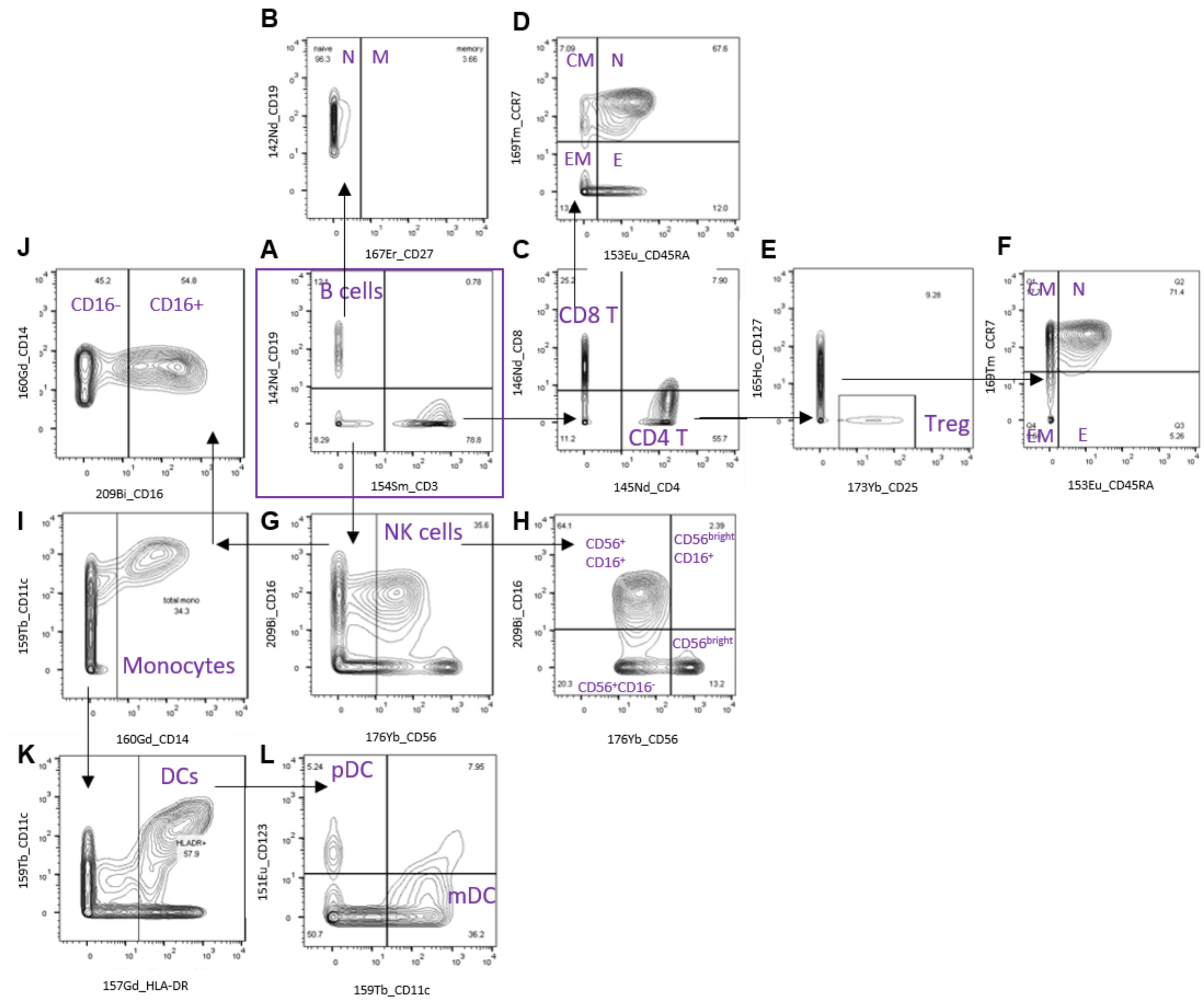

Fig. S7. Representative manual gating strategy. (A) Lineage markers CD3 and CD19 were firstly used to define CD3^{+} T cells and CD19^{+} B cells from the live, single cell file. **(B)** CD27 was used to determine memory (m) from naïve (n) B cells. **(C)** Within the CD3 T cell population, CD4 and CD8 T cells were identified. **(D)** CD8 T cells were classified into central memory (CM), naïve (N), effector memory (EM) and effector (E) populations based on CD45RA and CCR7 expression. **(E-F)** Regulatory CD4 T cells (Treg) were identified within the CD4 T cell population based on a CD127^{low}CD25^{+} profile. A 'not' gate was created and remaining CD4 T cells were classified into CM, N, EM and E populations based on CD45RA and CCR7 expression. **(G)** CD3^{-} CD19^{-}cells were assessed for expression of the NK cell marker CD56. **(H)** CD56^{+} NK cells were divided into their sub-populations based on both CD56 and CD16 expression; CD56^{+}CD16^{-}, CD56^{bright}CD16^{-}, CD56^{+}CD16^{+} CD56^{bright}CD16^{+}. **(I-J)** CD3^{-}CD19^{-}CD56^{-} cells were assessed for expression of the monocyte marker CD14, and further gated based on their expression of CD16. **(K-L)** CD3^{-}CD19^{-}CD56^{-}CD14^{-} cells were selected for positive expression of HLA-DR and subtyped into dendritic cell (DC) populations based on expression of CD11c (myeloid DCs (mDC)) and CD123 (plasmacytoid DCs (pDC)).

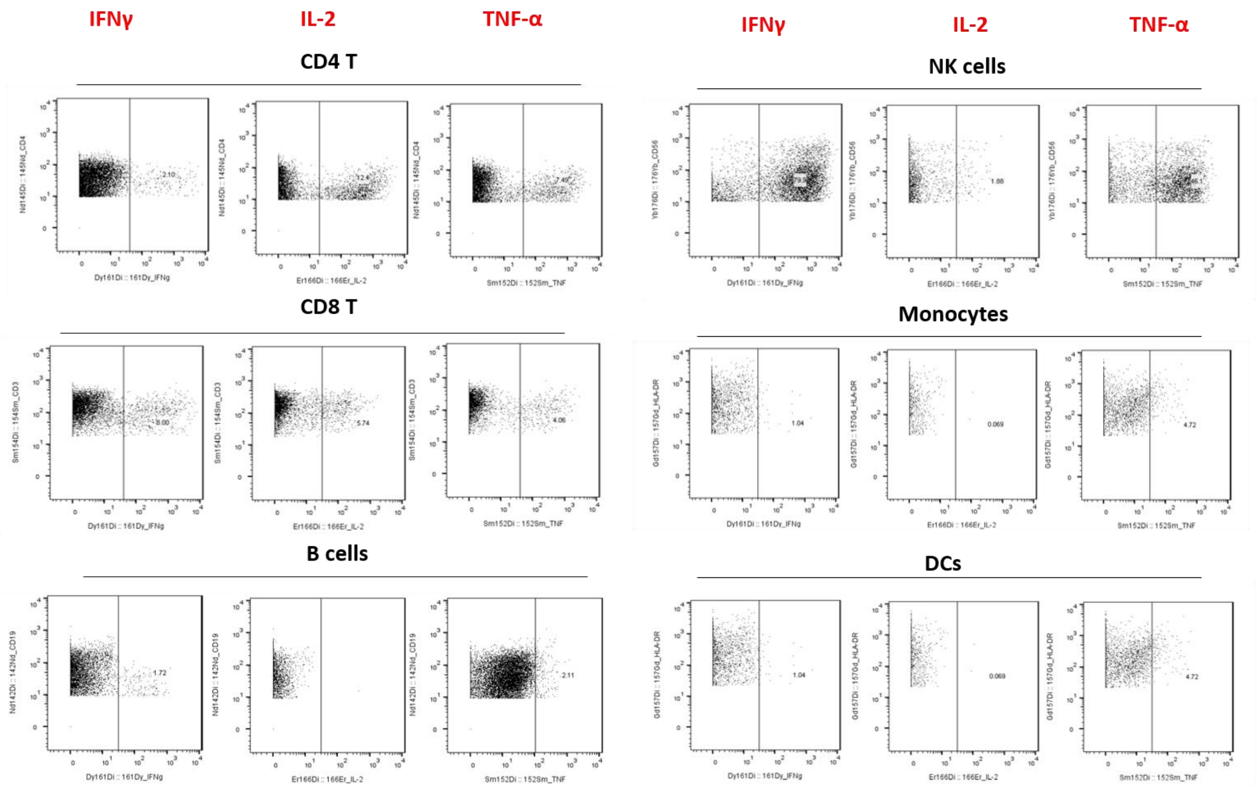

Fig. S8. Representative manual gating approach for analysis of IFNγ, IL-2 and TNFα following PMA/ionomycin stimulation in the major immune cell populations: CD4 T cells, CD8 T cells, B cells, NK cells, monocytes and dendritic cells (DCs).
