# Supplementary material for: Mass cytometry reveals cellular fingerprint associated with IgE+ peanut tolerance and allergy in early life
Source: Nat Commun. 2020 Feb 27;11:1091. doi: 10.1038/s41467-020-14919-4 (PMC7046671; doi:10.1038/s41467-020-14919-4)
Supplement: Supplementary file 3 — Reporting Summary [file 41467_2020_14919_MOESM3_ESM.pdf]

nature research

Corresponding author(s): Melanie Neeland

Last updated by author(s): Jan 20, 2020

Reporting Summary

Nature Research wishes to improve the reproducibility of the work that we publish. This form provides structure for consistency and transparency in reporting. For further information on Nature Research policies, see Authors & Referees and the Editorial Policy Checklist.

Statistics

For all statistical analyses, confirm that the following items are present in the figure legend, table legend, main text, or Methods section.

n/a	Confirmed	
☐	☒	The exact sample size (*n*) for each experimental group/condition, given as a discrete number and unit of measurement
☐	☒	A statement on whether measurements were taken from distinct samples or whether the same sample was measured repeatedly
☐	☒	The statistical test(s) used AND whether they are one- or two-sided<br>*Only common tests should be described solely by name; describe more complex techniques in the Methods section.*
☐	☒	A description of all covariates tested
☐	☒	A description of any assumptions or corrections, such as tests of normality and adjustment for multiple comparisons
☐	☒	A full description of the statistical parameters including central tendency (e.g. means) or other basic estimates (e.g. regression coefficient) AND variation (e.g. standard deviation) or associated estimates of uncertainty (e.g. confidence intervals)
☐	☒	For null hypothesis testing, the test statistic (e.g. *F*, *t*, *r*) with confidence intervals, effect sizes, degrees of freedom and *P* value noted<br>*Give P values as exact values whenever suitable.*
☒	☐	For Bayesian analysis, information on the choice of priors and Markov chain Monte Carlo settings
☒	☐	For hierarchical and complex designs, identification of the appropriate level for tests and full reporting of outcomes
☒	☐	Estimates of effect sizes (e.g. Cohen's *d*, Pearson's *r*), indicating how they were calculated

Our web collection on statistics for biologists contains articles on many of the points above.

Software and code

Policy information about availability of computer code

Data collection	no software was used for collection of data
Data analysis	FlowJo version 10.6<br>FlowSOM version 1.14.1<br>pheatmap version 1.0.12<br>U-MAP version 0.2.0.0<br>FlowStats version 3.40.1<br>ggplot2 version 3.1.0<br>R version 3.5.2

For manuscripts utilizing custom algorithms or software that are central to the research but not yet described in published literature, software must be made available to editors/reviewers. We strongly encourage code deposition in a community repository (e.g. GitHub). See the Nature Research guidelines for submitting code & software for further information.

Data

Policy information about availability of data

All manuscripts must include a data availability statement. This statement should provide the following information, where applicable:

- Accession codes, unique identifiers, or web links for publicly available datasets
- A list of figures that have associated raw data
- A description of any restrictions on data availability

The source data underlying all figures and supplemental data are provided as a Source Data file

October 2018

Field-specific reporting

Please select the one below that is the best fit for your research. If you are not sure, read the appropriate sections before making your selection.

☒ Life sciences ☐ Behavioural & social sciences ☐ Ecological, evolutionary & environmental sciences

For a reference copy of the document with all sections, see nature.com/documents/nr-reporting-summary-flat.pdf

Life sciences study design

All studies must disclose on these points even when the disclosure is negative.

Sample size

There were n=12 participants in each group (peanut allergic, peanut sensitized tolerant, healthy controls). Each sample underwent 3 in vitro stimulations. A total of 108 samples were analysed via mass cytometry.

Data exclusions

CD49b and LAG3 were excluded from manual and computational analysis due to limited expression across all samples. IL-10, IL-4 and IL-17A were excluded from manual and computational analysis due to limited expression across all samples.

Replication

findings cannot be replicated in these individuals as all cells were used for data collection

Randomization

samples were randomized prior to mass cytometry analysis and data collection

Blinding

investigators were blinded to food allergy outcome during data collection

Reporting for specific materials, systems and methods

We require information from authors about some types of materials, experimental systems and methods used in many studies. Here, indicate whether each material, system or method listed is relevant to your study. If you are not sure if a list item applies to your research, read the appropriate section before selecting a response.

Materials & experimental systems

n/a	Involved in the study	
☐	☒	Antibodies
☒	☐	Eukaryotic cell lines
☒	☐	Palaeontology
☒	☐	Animals and other organisms
☐	☒	Human research participants
☒	☐	Clinical data

Methods

n/a	Involved in the study	
☒	☐	ChIP-seq
☒	☐	Flow cytometry
☒	☐	MRI-based neuroimaging

Antibodies

Antibodies used

Metal label Specificity Clone / Catalogue
113In CD86 IT2.2 555662 BD
142Nd CD19 HIB19, 3142001B, Fluidigm
143Nd CD49b P1E6-C5, 359301 Biolegend
144Nd IL-4 MP4-25D2, 3144010B Fluidigm
145Nd CD4 RPA-T4, 3145001B Fluidigm
146Nd CD8 RPA-T8, 3162015B Fluidigm
147Sm CD20 2H7, 3147001BFluidigm
148Nd CD38 HB-7, BD
149Sm CCR4 205410, 3149003A Fluidigm
150Nd LAG3 874501, 3150016B Fluidigm
151Eu CD123 6H6, 3151001B Fluidigm
152Sm TNF Mab11, 3152002B Fluidigm
153Eu CD45RA HI100, 3153001B Fluidigm
154Sm CD3 UCHT1, 3154003B Fluidigm
155Gd CD28 L283, 340975 BD,
157Gd HLA-DR G46-6, 555810 BD
158Gd CD33 WM53, 3158001B Fluidigm
159Tb CD11c Bu15, 3159001B Fluidigm
160Gd CD14 M5E2, 3160001B Fluidigm
161Dy IFNg 4S.B4, 147319-85 eBioscience
162Dy CD69 FN50, 3162001B Fluidigm
163Dy CXCR3 G025H7, 3163004B Fluidigm
164Dy IL-17 N49-853, 3164002B Fluidigm

	165Ho CD127 A019D5, 3165008B Fluidigm<br>166Er IL-2 MQ1-17h12, 3166002B Fluidigm<br>167Er CD27 L128, 3167006B Fluidigm<br>168Er CD40L 24-31, 3168006B Fluidigm<br>169Tm CCR7 15053, MAB197 R&D Systems,<br>171Yb IL-10 JES3-9D7, Biolegend<br>173Yb CD25 M-A251, 555430 BD,<br>176Yb CD56 NCAM16.2, 3176008B Fluidigm<br>209Bi CD16 3G8, 3209002B Fluidigm
Validation	Each lot of conjugated antibody is quality control tested by CyTOF analysis of stained cells using the appropriate positive and negative cell staining and/or activation controls.

Human research participants

Policy information about studies involving human research participants

Population characteristics	Blood samples from one year old infants with peanut allergy, peanut sensitization and healthy controls were analyzed. 42% of healthy controls were male, 58% of peanut sensitization group were male and 67% of the peanut allergic group were male. Information on ancestry, family history of allergy, and other allergic conditions are specified in Table 1 of the manuscript.
Recruitment	Patients were recruited as per the HealthNuts Population Cohort study profile (Osborne et al., J. Allergy Clin Immunol 2011.)
Ethics oversight	Approval to conduct the HealthNuts study was obtained from the Victorian State Government Office for Children (reference no. CDF/07/492), the Victorian State Government Department of Human Services (reference no. 10/07), and the Royal Children's Hospital Human Research Ethics Committee (reference no. 27047).

Note that full information on the approval of the study protocol must also be provided in the manuscript.
